# Metabolic biomarkers of neonatal sepsis: identification using metabolomics combined with machine learning

**DOI:** 10.3389/fcell.2024.1491065

**Published:** 2024-10-21

**Authors:** Zhaonan Bian, Xinyi Zha, Yanru Chen, Xuting Chen, Zhanghua Yin, Min Xu, Zhongxiao Zhang, Jihong Qian

**Affiliations:** ^1^ Department of Neonatology, Xinhua Hospital, Shanghai Jiaotong University School of Medicine, Shanghai, China; ^2^ Hongqiao International Institute of Medicine, Tongren Hospital, Shanghai Jiaotong University School of Medicine, Shanghai, China

**Keywords:** sepsis, infant, metabolomics, biomarker, meaching learning

## Abstract

**Background:**

Sepsis is a common disease associated with neonatal and infant mortality, and for diagnosis, blood culture is currently the gold standard method, but it has a low positivity rate and requires more than 2 days to develop. Meanwhile, unfortunately, the specific biomarkers for the early and timely diagnosis of sepsis in infants and for the determination of the severity of this disease are lacking in clinical practice.

**Methods:**

Samples from 18 sepsis infants with comorbidities, 25 sepsis infants without comorbidities, and 25 infants with noninfectious diseases were evaluated using a serum metabolomics approach based on liquid chromatography‒mass spectrometry (LC‒MS) technology. Differentially abundant metabolites were screened via multivariate statistical analysis. In addition, least absolute shrinkage and selection operator (LASSO) and support vector machine recursive feature elimination (SVM-RFE) analyses were conducted to identify the key metabolites in infants with sepsis and without infections. The random forest algorithm was applied to determine key differentially abundant metabolites between sepsis infants with and without comorbidities. Receiver operating characteristic (ROC) curves were generated for biomarker value testing. Finally, a metabolic pathway analysis was conducted to explore the metabolic and signaling pathways associated with the identified differentially abundant metabolites.

**Results:**

A total of 189 metabolites exhibited significant differences between infectious infants and noninfectious infants, while 137 distinct metabolites exhibited differences between septic infants with and without comorbidities. After screening for the key differentially abundant metabolites using LASSO and SVM-RFE analyses, hexylamine, psychosine sulfate, LysoPC (18:1 (9Z)/0:0), 2,4,6-tribromophenol, and 25-cinnamoyl-vulgaroside were retained for the diagnosis of infant sepsis. ROC curve analysis revealed that the area under the curve (AUC) was 0.9200 for hexylamine, 0.9749 for psychosine sulfate, 0.9684 for LysoPC (18:1 (9Z)/0:0), 0.7405 for 2,4,6-tribromophenol, 0.8893 for 25-cinnamoyl-vulgaroside, and 1.000 for the combination of all metabolites. When the septic infants with comorbidities were compared to those without comorbidities, four endogenous metabolites with the greatest importance were identified using the random forest algorithm, namely, 12-oxo-20-trihydroxy-leukotriene B4, dihydrovaltrate, PA (8:0/12:0), and 2-heptanethiol. The ROC curve analysis of these four key differentially abundant metabolites revealed that the AUC was 1 for all four metabolites. Pathway analysis indicated that phenylalanine, tyrosine, and tryptophan biosynthesis, phenylalanine metabolism, and porphyrin metabolism play important roles in infant sepsis.

**Conclusion:**

Serum metabolite profiles were identified, and machine learning was applied to identify the key differentially abundant metabolites in septic infants with comorbidities, septic infants without comorbidities, and infants without infectious diseases. The findings obtained are expected to facilitate the early diagnosis of sepsis in infants and determine the severity of the disease.

## Background

The immature neonatal adaptive immune system causes newborns to rely primarily on their innate immune system to combat the environmental microorganisms encountered after birth. Consequently, newborns are highly susceptible to infections, which leads to a higher mortality rate of approximately 3 million neonatal deaths occurring annually across the world, among which 40% of fatalities occur due to infectious diseases (Liu et al., 2012). Neonatal sepsis is responsible for 15% of perinatal deaths (Lawn et al., 2014). According to a global data report from 2018, nearly three million cases of sepsis in newborns are reported each year, with a mortality rate ranging from 11% to 19% (Fleischmann-Struzek et al., 2018). Premature infants are at an even greater risk of developing sepsis and have higher mortality rates than full-term newborns. An analysis of Chinese data between 2015 and 2018 revealed that the incidence rate of neonatal sepsis in infants born before 34 weeks was 9.7 per thousand cases, with a case fatality rate of 22.6% (Jiang et al., 2019).

In clinical practice, the presentations of neonatal sepsis frequently deviate from the typical presentations of sepsis. Blood culture is currently the gold standard for establishing a diagnosis of neonatal sepsis, although it requires a minimum of 24–48 h. In addition, due to factors such as blood collection volume and culture methods, the positive rate of blood culture is often low in clinical practice. Therefore, relying solely on blood culture results often leads to delayed or missed diagnoses (De Rose et al., 2021). Due to the lack of unique biomarkers to date, the early identification of neonatal sepsis relies primarily on clinical laboratory tests such as C-reactive protein (CRP), procalcitonin (PCT), and white blood cell (WBC) (Bunduki and Adu-Sarkodie, 2020; Boscarino et al., 2023), which are not feasible to simultaneously achieve high specificity and high sensitivity. CRP is widely recognized as a biomarker in the diagnosis of neonatal sepsis. However, its specificity and sensitivity vary significantly across different clinical studies due to the application of diverse cut-off values. Furthermore, CRP has a short half-life limitation and may also be elevated in non-infected neonates (Cantey and Bultmann, 2020; Jyoti et al., 2021). Compared to CRP, PCT is considered as a better biomarker for neonatal sepsis diagnosis because of its greater sensitivity and accuracy (Gopal et al., 2023). Unfortunately, PCT spontaneously increases after birth in healthy neonates, and there are more obvious changes in PCT in preterm neonates; moreover, noninfective perinatal circumstances may also increase the serum PCT concentration, limiting the clinical use of PCT (Boscarino et al., 2023). WBC has very poor sensitivity, and similar WBC values were found in healthy neonates, infected neonates and neonates with other diseases (Hornik et al., 2012). The early diagnosis of neonatal sepsis and the assessment of the severity of this condition could facilitate the planning of a treatment plan designed precisely according to patient requirements while enhancing overall disease outcomes and prognosis. This would also prevent the occurrence of potential adverse effects on neonatal immune function and growth due to the excessive use of antibiotics (Hou et al., 2022). Thus, it is necessary to identify excellent biomarkers that could facilitate an improved diagnosis of neonatal sepsis.

According to current research, a robust correlation exists between inflammation and metabolism. In sepsis, the body’s metabolic homeostasis is disrupted, which activates the hypothalamic‒pituitary‒adrenal axis, resulting in increased secretion of cortisol and catecholamines and extensive release of cytokines. These changes subsequently impact various metabolic pathways, including glycolysis, the tricarboxylic acid cycle, lipid metabolism, and amino acid metabolism (Eckerle et al., 2017; Mierzchala-Pasierb et al., 2020). Metabolomics allows for the simultaneous detection of multiple metabolic molecules, thereby enabling a further comprehensive assessment of the body’s condition during infection and inflammation. In recent years, research on the biological changes in metabolites in children with severe infections has improved our knowledge of the complex and dynamic mechanisms underlying sepsis (Fanos et al., 2014; Sarafidis et al., 2017; Bjerkhaug et al., 2021). Mardegan et al. (2021) utilized untargeted and targeted metabolomics to compare metabolic changes between children with early-onset neonatal sepsis and children without infection. They found significant disruption of glutathione and tryptophan metabolic pathways in the case group, and metabolites of the glutathione and tryptophan pathways are promising new biomarkers of neonatal sepsis. Wang et al. (2023) reported significant alterations in lipid metabolism in infants with sepsis, with certain metabolites reported to exhibit substantial potential for the early detection of this condition. However, these authors failed to address the metabolic variances among infants with varying degrees of disease severity. Early assessment of disease severity holds paramount importance in developing strategies for determining the prognosis and management of this condition in infants or newborn patients.

In this context, the present study aimed to identify differentially abundant metabolites in serum samples from infants with septicemia using liquid chromatography/mass spectrometry (LC/MS) and then applied machine learning techniques to identify the key differentially abundant metabolites. Machine learning algorithms can efficiently process complex metabolomics data and filter out representative metabolites to differentiate between different groups. The objective of this study was to explore and identify excellent biomarkers for the early diagnosis of neonatal sepsis and to determine the severity of this condition for developing precise treatment plans for these children to improve their prognosis.

## Methods

### Patient recruitment

A total of 68 patients admitted to Xinhua Hospital between July 2022 and July 2023 were enrolled in the present study. Infants with sepsis caused by bacterial infections and only four patients with sepsis were admitted to the neonatal intensive care unit. The comorbidity group (SI) comprised 18 children who were diagnosed with sepsis along with comorbidities, including shock, suppurative meningitis, coagulation disorder, and urinary tract infection. The sepsis group (INF) included 25 children who were diagnosed with sepsis without complications. All of these children exhibited signs of infection and were confirmed to have sepsis through a series of follow-up laboratory tests, including positive blood cultures or elevated CRP and PCT levels (Shane et al., 2017). In addition, 25 children without any infectious disease were enrolled in the study to serve as the control group (CON). All patients were less than 3 months of age and were matched for sex, weight, and age on the day of enrollment. The exclusion criteria for participation in the study were as follows: 1) infants who had received antibiotics for more than 7 days prior to admission; 2) infants who had received total parenteral nutrition support for more than 7 days prior to admission; 3) infants diagnosed with infantile cholestasis or congenital heart disease; 4) infants with confirmed or suspected congenital metabolic diseases; and 5) mothers with a history of gestational diabetes, intrahepatic cholestasis, or abnormal liver function. The study procedure was approved by the Ethics Committee of Xinhua Hospital (approval number XHEC-C-2020–091–1). Informed written consent was obtained from the parents of all enrolled patients prior to commencing the study.

### Sample collection and preparation

Peripheral blood (2 mL) samples were collected simultaneously under aseptic conditions, and within 2 h, each of the collected peripheral blood samples was centrifuged at 3,000 rpm for 15 min at 4°C to obtain serum. The resulting supernatant was then transferred to a 1.5 mL EP tube and stored at −80°C until use. The sample preparation process involved the addition of approximately 100 µL of serum to a methanol solution (containing 5 μg/mL L-2-chloro-phenylalanine as the internal standard) at a ratio of 1:3, followed by 2 min of vortexing. Subsequently, centrifugation was performed at 13,000 rpm and 4°C for 10 min, and 200 µL of the resulting supernatant was collected. Finally, equal volumes of serum from each sample were pooled to obtain a quality control (QC) sample.

### LC/MS analysis

Ultimate 3,000 ultra-high performance liquid chromatography coupled with Thermo Orbitrap Elite mass spectrometry was employed for the LC/MS analysis. The column employed was Kinetex C18 (100 mm × 2.1 mm, 1.9 µm). The mobile phase comprised solution A (0.1% formic acid solution) and solution B (acetonitrile in 0.1% formic acid). The flow rate is set to 0.4 mL/min. A column temperature of 25°C was maintained. The post time was set to 5 min, and the sample volume of 3 µL was used. In the mass spectrometry analysis, both positive ion mode and negative ion mode were used with specific optimization parameters. In the positive ion mode, the following parameters were used: heater temperature, 300°C; sheath gas flow rate, 45 arb; aux gas flow rate, 15 arb; sweep gas flow rate, 1 arb; spray voltage, 3.0 kV; capillary temperature, 350°C; S-Lens RF level, 30%. The scan range was 200 to 1,500. In the negative ion mode, the following parameters were used: heater temperature, 300°C; sheath gas flow rate, 45 arb; aux gas flow rate, 15 arb; sweep gas flow rate, 1 arb; spray voltage, 2.5 kV; capillary temperature, 350°C; S-Lens RF level, 60%. The scan range was 200 to 1,500. The compound components in the samples, as detected by LC/MS, were extracted and preprocessed using Compound Discovery 3.0 software from Thermo Scientific, United States. This process involved baseline filtering, peak identification and integration, retention time correction, peak alignment, and mass spectrometry fragment assignment. Subsequently, the data were further edited in Excel software. After preprocessing, a data matrix was obtained that contained information on the mass-to-charge ratio (m/z), retention time (RT), and normalized peak intensity of the metabolites. This matrix was used for the final analysis to deduce the structural information of the compounds.

### Machine learning

The machine learning analysis was conducted using R software (version 4.4.0; AutoDesk, United States). Least absolute shrinkage and selection operator (LASSO) screening to identify the characteristic metabolites was performed using the glmnet package in R. Feature screening with support vector machine recursive feature elimination (SVM-RFE) was conducted using the e1071 package and doParallel package. The random forest algorithm was applied to rank and screen the important metabolites. Finally, the results were visualized using the plot function in R.

### Statistical analysis

The clinical data were recorded and edited using Microsoft Excel and then analyzed using SPSS Statistics 26 (version 26.0.0.0, IBM, United States). The metabolomics results were detected through orthogonal corrected partial least squares discriminant analysis (OPLS-DA) and principal component analysis (PCA) performed using Simca-P software (version 11.0; Umetrics, Sweden). The OPLS-DA permutation test was conducted using positive and negative ion models with 200-fold cross-validation. The metabolites with variable importance in projection (VIP) > 1 and *P* < 0.05 were considered for the follow-up analysis. Pathway analysis of the differentially abundant metabolites was performed using the MetaboAnalyst online analysis website. Group comparisons were conducted for the clinical data through t‒test, analysis of variance (ANOVA) and Mann‒Whitney U test. Categorical variables were compared between the groups using chi-squared tests or Fisher’s exact tests. In all analyses, *p* < 0.05 was set as the threshold for statistical significance. The violin plots and ROC curves were generated using GraphPad Prism 8 (version 8.0.1, GraphPad Software, United States).

## Results

### Characteristics of the study population

A total of 68 children were enrolled in the present study, including 18 children with concurrent infections and complications (SI), 25 children with infection and no complications (INF), and 25 children without any infection (CON). Table 1 summarizes the characteristics of all 68 study participants. Among the 43 infected children, 15 had positive blood culture results, including 9 in the SI group and 6 in the INF group. Among the 18 patients in the SI group, 13 had urinary bacteria, 5 had suppurative meningitis, and 1 had coagulation dysfunction. No significant differences were noted among the three groups regarding the sex composition ratio, age at blood collection, preterm birth rate, gestational age, or birth weight (*P* > 0.05). Laboratory tests revealed that the neutrophil percentage (N%) and the levels of CRP and PCT were significantly higher in both the SI group and INF group than in the control group (*P* < 0.05). In addition, the WBC was higher in the INF group than in the SI group (*P* < 0.05).

**TABLE 1:** Characteristics of the study participants (n = 68).

Descriptive variable	SI(n = 18)	INF(n = 25)	CON(n = 25)	*X* ^*2*^ */Z/F*	*P*
Male sex (%)	14 (77.8)	16 (64.0)	13 (52.0)	0.22	0.242
Age [days]	36 [49]	25 [56]	32 [39]	2.02	0.364
Premature (%)	2 (11.1)	2 (8.0)	3 (12.0)	—	1.000
Gestational age (weeks)	38.5 ± 1.7	38.8 ± 1.2	38.5 ± 1.4	0.39	0.676
Birth weight (g)	3,126 ± 518	3,345 ± 385	3,235 ± 388	1.41	0.253
Weight^1^ [g]	4,510 [2,158]	4,390 [2,135]	4,100 [1745]	0.61	0.738
Breastfed (%)	5 (27.8)	6 (24.0)	10 (40.0)	0.45	0.502
N% (%)	54.6 ± 14.1	59.0 ± 17.6	26.62 ± 12.1^a,b^	33.91	<0.01
WBC [10^9^/L]	10.29 [9.23]	17.8 [7.59]^a^	8.28 [3.96]^b^	23.11	<0.01
CRP [mg/L]	49 [68]	38 [58]	0 [0]^a,b^	48.10	<0.01
PCT [ng/mL]	0.67 [1.72]	3.04 [13.57]	0.08 [0.05]^a,b^	42.69	<0.01

The normally distributed numerical data are reported as means±standard deviations. The non-normally distributed data are reported as median [interquartile range]. Categorical data are reported as the number of cases (percentage).

The presence of “a” indicates that the observed difference is statistically significant compared to the SI, group, while the presence of “b” indicates that the observed difference is statistically significant compared to the INF, group.

### Metabolomic profiling of serum samples

**FIGURE 1:**
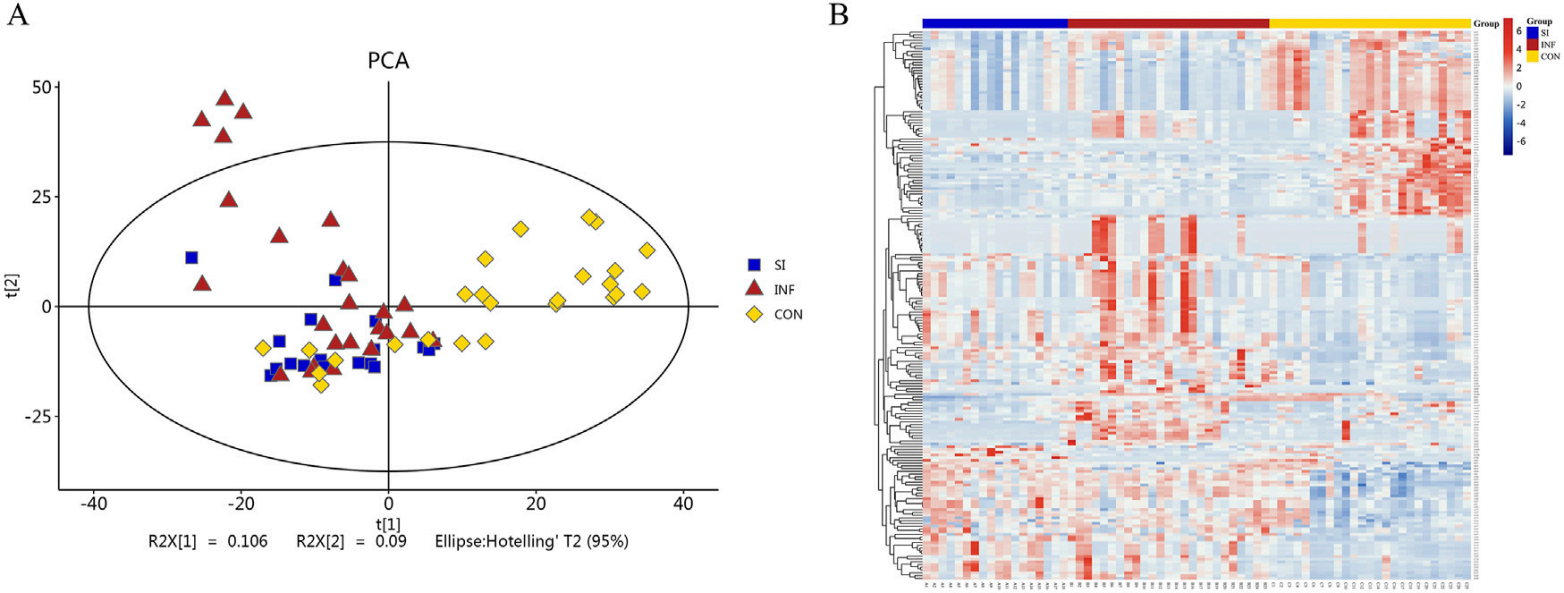
PCA plots and heatmap of the identified differential metabolites. **(A)** PCA plots of the differential metabolites among the three groups. **(B)** Heatmap illustrating the differential metabolites among the three groups.

**FIGURE 2:**
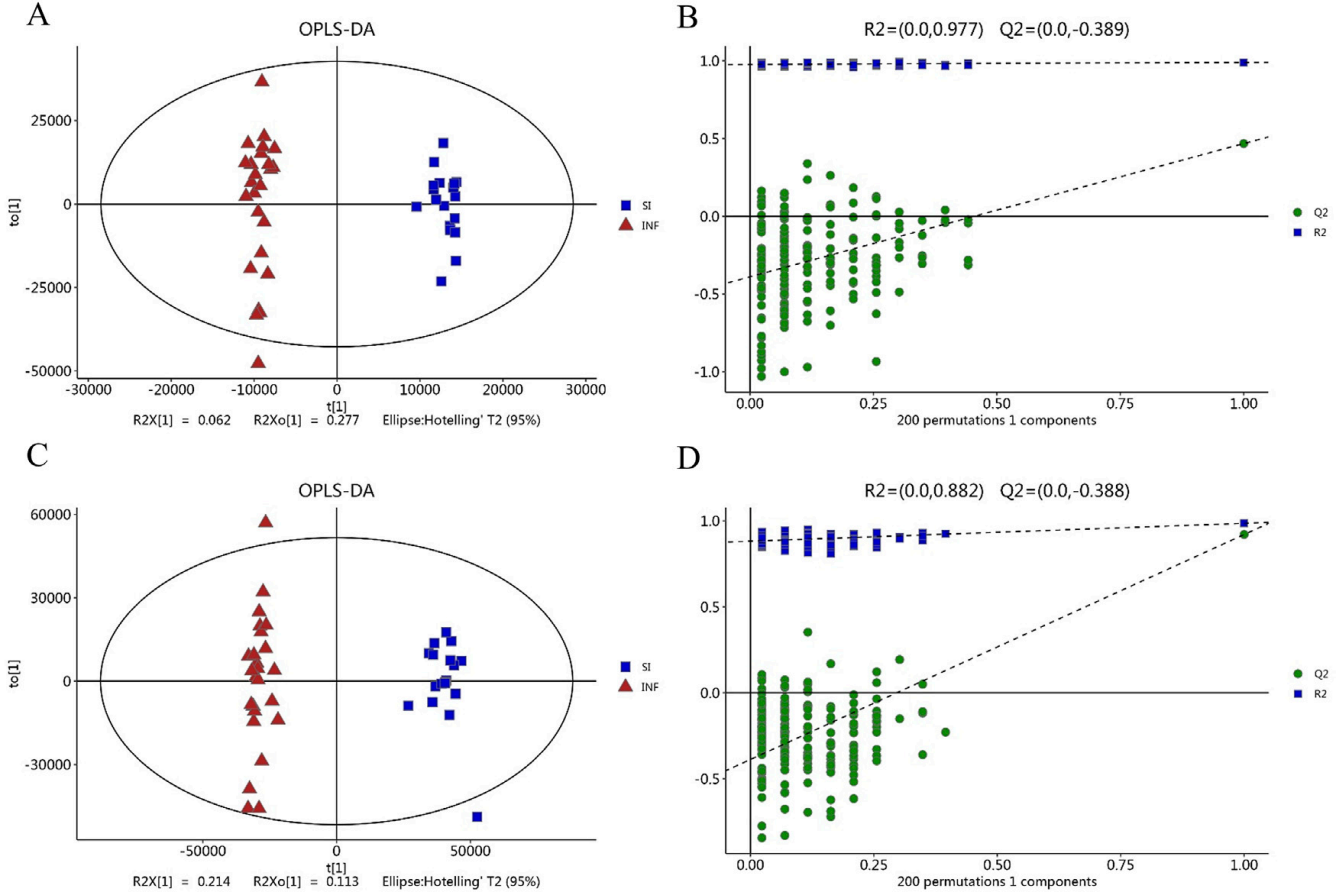
The score plot based on OPLS-DA model. **(A)** OPLS-DA score map in negative ion mode; **(B)** Permutation test with OPLS-DA model in negative mode; **(C)** OPLS-DA score map in positive ion mode; **(D)** Permutation test with the OPLS-DA model in positive ion mode.

The serum samples were analyzed based on untargeted metabolomics technology and the obtained data were processed. In negative ion mode, we extracted a total of 2,491 features, while in positive ion mode, we extracted 4,349 features. The differences in the metabolite detection results among the three groups were described through principal component analysis and a heatmap (Figure 1). PCA revealed significant clustering within the three groups, with a distinct separation between the DIS group (including the SI group and the INF group) and the CON group. OPLS-DA was subsequently performed to identify the metabolites that resulted in these significant differences. The OPLS-DA score maps of the SI group and the INF group were generated using positive and negative ion models with 200-fold cross-validation (Figure 2). In the resulting OPLS-DA score map, a distinct separation could be noted between the groups. The cumulative R2X = 0.67\R2Y = 0.997\Q2 = 0.468 in negative ion mode and R2X = 0.47\R2Y = 0.882\Q2 = 0.921 in positive ion mode indicated that the model was reliable. Next, using the screening criteria of *P* < 0.05 and VIP > 1 by OPLS-DA model, 189 metabolites exhibiting significant differences between the DIS group and the CON group were identified. These metabolites were then analyzed further, revealing 137 key metabolites exhibiting variations between the SI group and the INF group.

### Key differentially abundant metabolites between the DIS group and the CON group

**FIGURE 3:**
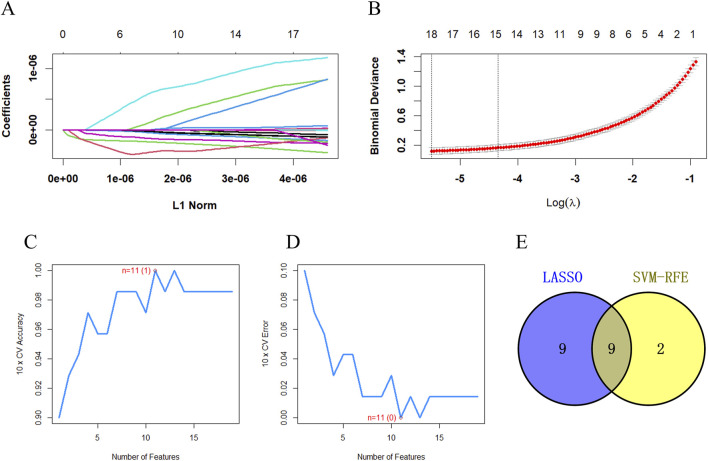
The key differentially abundant metabolites between the DIS group and the CON group identified using LASSO and SVM-RFE. **(A)** The key differentially abundant metabolites identified using LASSO; **(B)** LASSO cross-validation diagram; **(C)** The key differentially abundant metabolites identified through SVM-RFE along with the SVM-RFE error diagram; **(D)** SVM-RFE accuracy diagram; **(E)** Venn diagram depicting the diagnostic biomarkers obtained through the intersection of the results from the two algorithms.

LASSO and SVM-RFE were employed in a total of 189 distinct metabolites between the DIS group and the CON group. According to the results of LASSO, 18 significantly differentially abundant metabolites demonstrating minimal error in the cross-validation were retained as predictors (Figures 3A, B). The SVM-RFE screening identified a total of 11 distinct metabolites with minimal cross-validation error and the highest accuracy (Figures 3C, D). Subsequently, the intersection of the results from these two machine learning algorithms yielded a final set of nine differentially abundant metabolites, which were then subjected to further analysis (Figure 3E; Table 2). After excluding the exogenous metabolites, five metabolites were retained as biomarkers for neonatal sepsis diagnosis: hexylamine, psychosine sulfate, LysoPC (18:1 (9Z)/0:0), 2,4,6-tribromophenol, and 25-cinnamoyl-vulgaroside.

**TABLE 2:** Key differentially abundant metabolites between the DIS group and the CON group.

HMDB_ID	Name	m/z	RT [min]	Endogenous	*P*
HMDB0032323	Hexylamine	102.13	1.59	YES	<0.005
HMDB0013046	Psychosine sulfate	271.66	8.99	YES	<0.001
HMDB0010397	LysoPC(20:5 (5Z,8Z,11Z,14Z,17Z)/0:0)	542.32	8.86	NO	<0.001
HMDB0002815	LysoPC(18:1 (9Z)/0:0)	522.35	8.89	YES	<0.001
HMDB0014118	Trifluoroacetic acid	112.99	1.01	NO	<0.001
HMDB0014838	Eplerenone	413.20	7.02	NO	<0.001
HMDB0029642	2,4,6-Tribromophenol	326.76	1.25	YES	<0.001
HMDB0041367	25-Cinnamoyl-vulgaroside	565.33	9.12	YES	<0.001
HMDB0038714	Kelampayoside A	238.08	17.27	NO	<0.001

**FIGURE 4:**
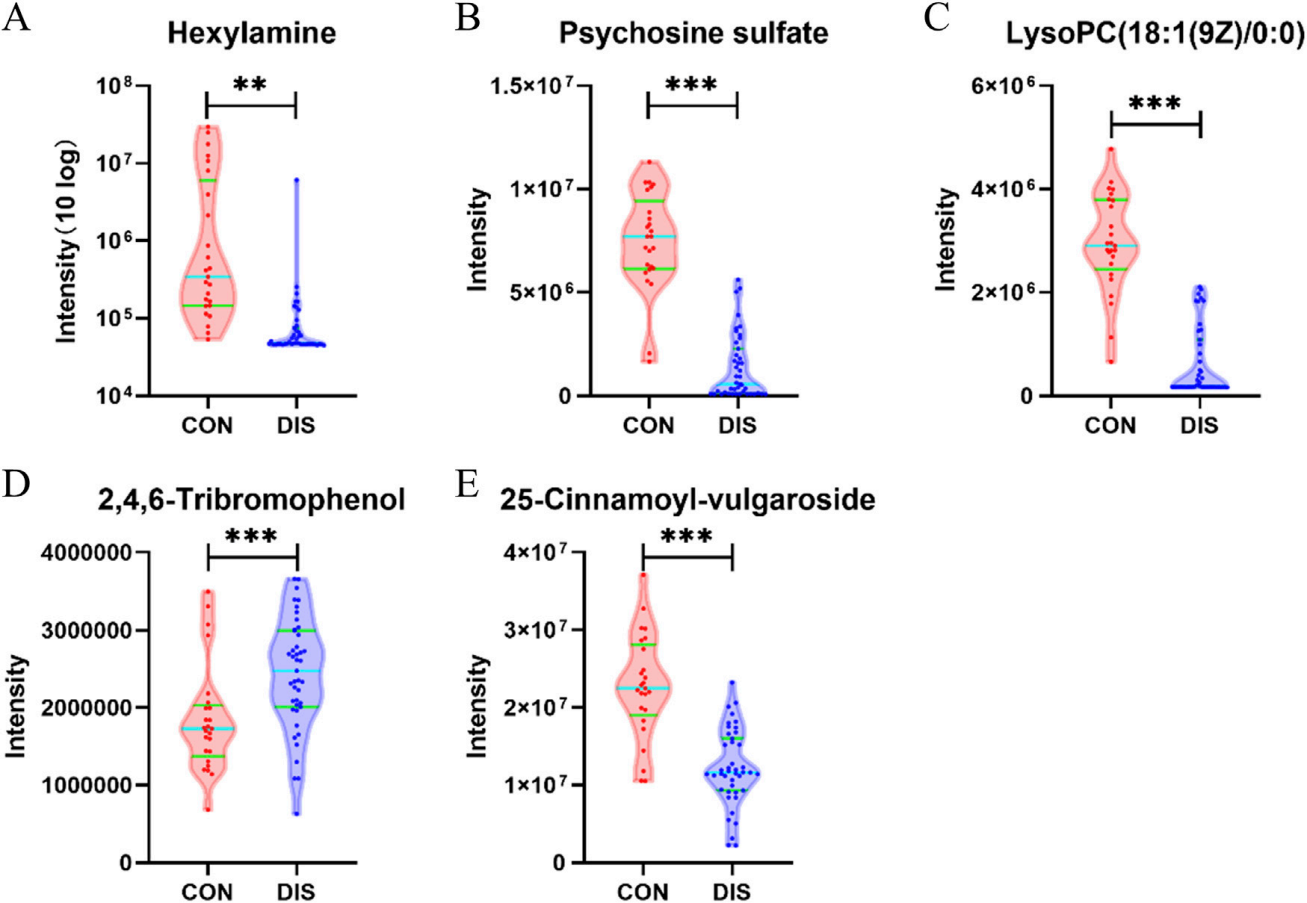
Violin plots depicting the key differentially abundant metabolites between the DIS group and the CON group (*p < 0.05; **p < 0.01; ***p < 0.001). **(A)** Hexylamine; **(B)** Psychosine sulfate; **(C)** LysoPC (18:1 (9Z)/0:0); **(D)** 2,4,6-Tribromophenol; **(E)** 25-Cinnamoyl-vulgaroside.

Compared with the CON group, the DIS group exhibited a significant increase in the serum levels of 2,4,6-tribromophenol. Conversely, a notable decrease was noted in hexylamine, psychosine sulfate, LysoPC (18:1 (9Z)/0:0), and 25-cinnamoyl-vulgaroside (Figure 4).

### ROC analysis of the key differentially abundant metabolites between the DIS group and the CON group

**FIGURE 5:**
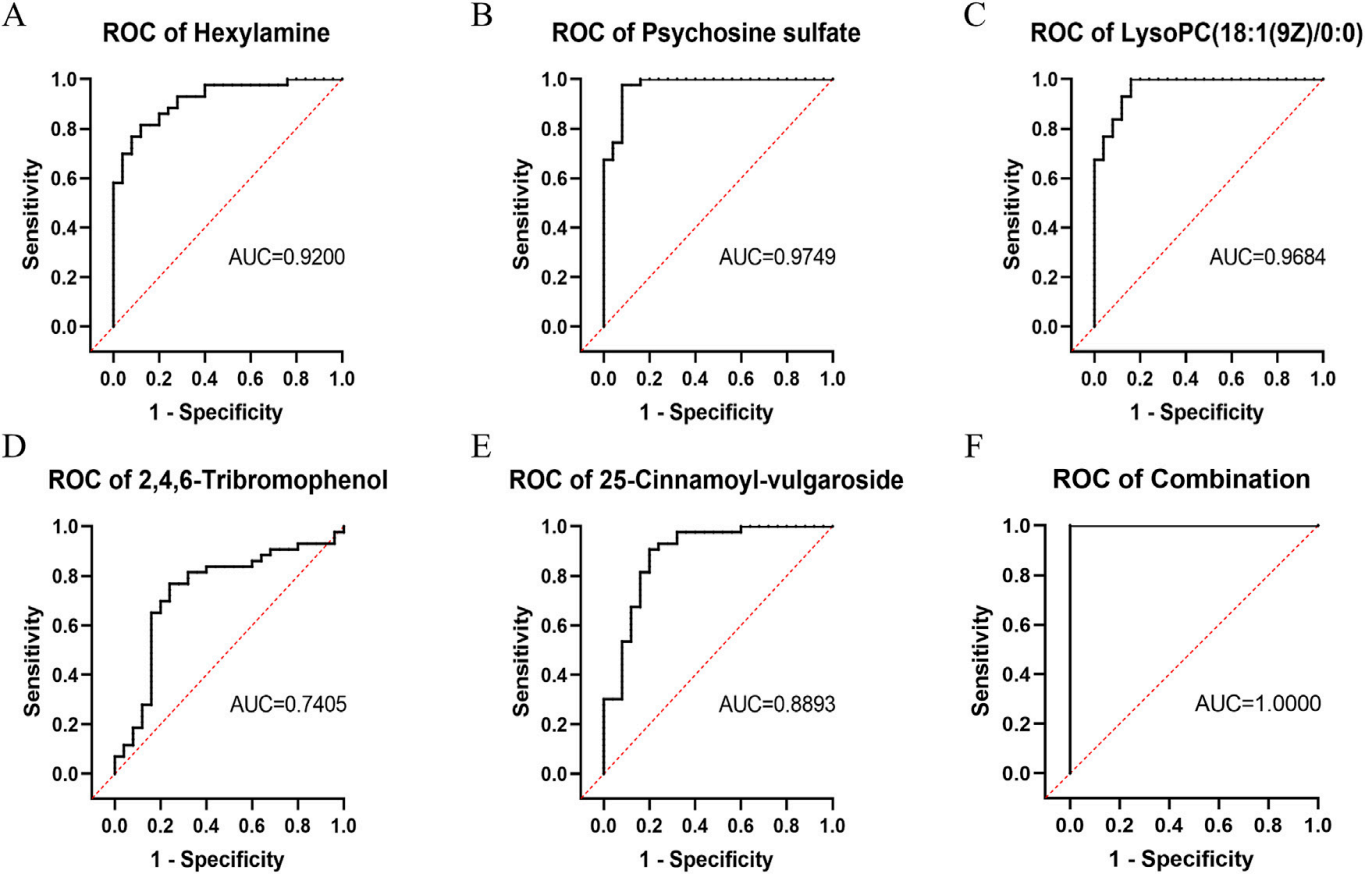
ROC curves of the key differentially abundant metabolites between the DIS group and the CON group. **(A)** Hexylamine; **(B)** Psychosine sulfate; **(C)** LysoPC (18:1 (9Z)/0:0); **(D)** 2,4,6-Tribromophenol; **(E)** 25-Cinnamoyl-vulgaroside; **(F)** Combination of all five metabolites.

ROC analysis was performed for the five key differentially abundant metabolites identified in the previous step. Hexylamine, psychosine sulfate, and LysoPC (18:1 (9Z)/0:0) exhibited area under the curve (AUC) values > 0.9. The AUC for 25-cinnamoyl-vulgaroside was > 0.8, while that for 2,4,6-tribromophenol was > 0.7. Notably, when all five metabolites were considered collectively, the combined AUC was 1 (Figure 5).

### Key differentially abundant metabolites between the SI group and the INF group

**FIGURE 6:**
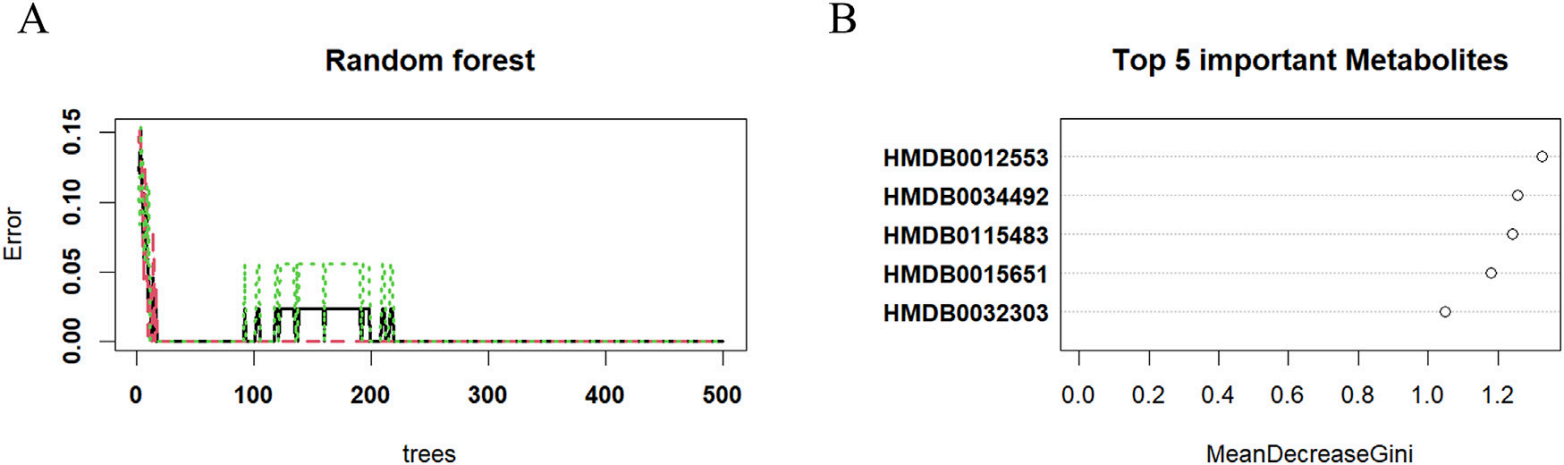
The key differentially abundant metabolites identified by applying the random forest algorithm between the SI group and the INF group. **(A)** The Random Forest error diagram; **(B)** Five metabolites with the highest importance.

The random forest algorithm was applied to identify key differentially abundant metabolites from 137 differentially abundant metabolites between the SI group and the INF group. First, the number of trees was determined, and it was observed that upon reaching 250 trees, the model’s error rate stabilized. Therefore, 250 decision trees were used for model construction (Figure 6A). Using this model comprising 250 decision trees, five key differentially abundant metabolites with the highest importance were identified between the two groups (Figure 6B). After excluding the exogenous metabolites, four key differentially abundant metabolites were retained as biomarkers capable of distinguishing the SI group from the INF group: 12-oxo-20-trihydroxy-leukotriene B4, dihydrovaltrate, PA (8:0/12:0), and 2-heptanethiol (Table 3).

**TABLE 3:** Key differentially abundant metabolites between the SI group and the INF group.

HMDB_ID	Name	m/z	RT [min]	Endogenous	*P*
HMDB0012553	12-Oxo-20-trihydroxy-leukotriene B4	383.20	11.10	YES	<0.001
HMDB0034492	Dihydrovaltrate	425.22	11.57	YES	<0.001
HMDB0115483	PA (8:0/12:0)	481.29	17.33	YES	<0.001
HMDB0015651	Isometheptene	142.16	4.24	NO	<0.005
HMDB0032303	2-Heptanethiol	133.11	2.80	YES	<0.001

### Analysis of the key differentially abundant metabolites between the SI group and the INF group

**FIGURE 7:**
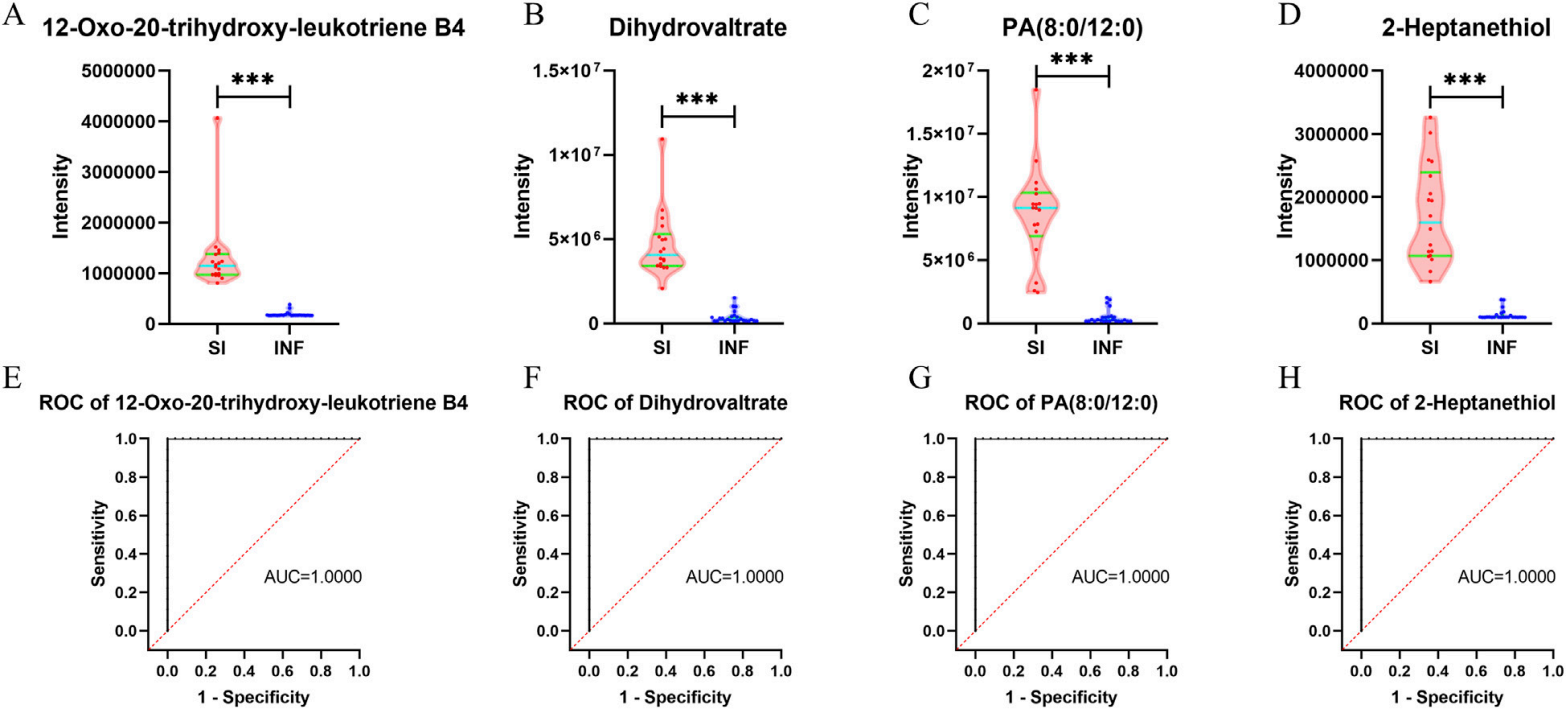
Violin plots and ROC curves for the identified key differentially abundant metabolites between the SI group and the INF group (*p < 0.05; **p < 0.01; ***p < 0.001). **(A)** Violin plot for 12-oxo-20-trihydroxy-leukotriene B4; **(B)** Violin plot for dihydrovaltrate; **(C)** Violin plot for PA (8:0/12:0); **(D)** Violin plot for 2-heptanethiol; **(E)** ROC curve for 12-oxo-20-trihydroxy-leukotriene B4; **(F)** ROC curve for dihydrovaltrate; **(G)** ROC curve for PA (8:0/12:0); **(H)** ROC curve for 2-heptanethiol.

Among the four key differentially abundant metabolites between the SI group and the INF group, 12-oxo-20-trihydroxy-leukotriene B4, dihydrovaltrate, PA (8:0/12:0), and 2-heptanethiol exhibited significant upregulation in the SI group compared to the INF group (Figures 7A–D). ROC analysis of these four key differentially abundant metabolites revealed that AUC for all four metabolites was 1 (Figures 7E–H).

### Metabolite pathway analysis

**FIGURE 8:**
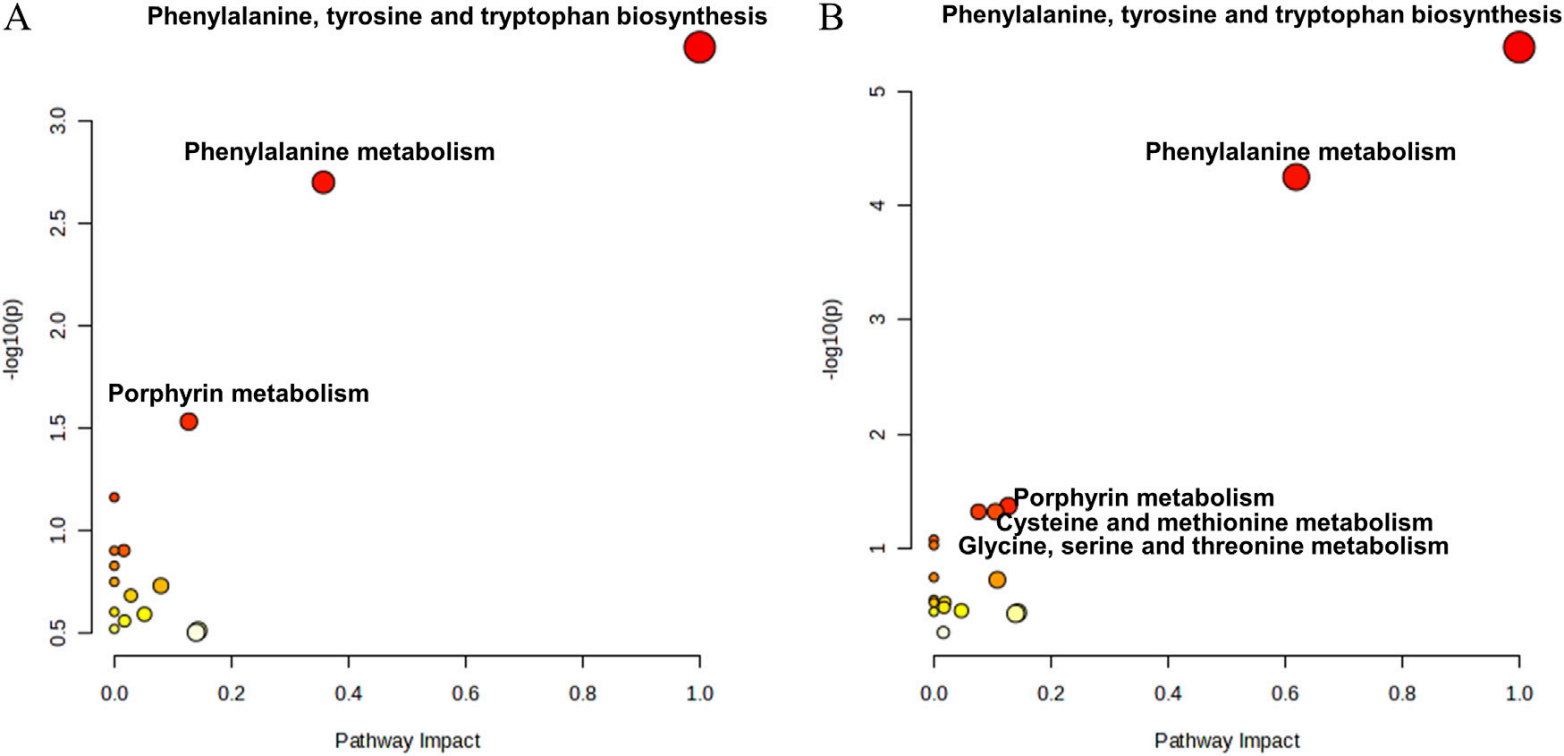
The bubble diagram for the results of KEGG pathway analysis. **(A)** Results of the pathway analysis between the DIS group and the CON group; **(B)** Results of the pathway analysis between the SI group and the INF group.

Kyoto Encyclopedia of Genes and Genomes (KEGG) pathway analysis of the identified differentially abundant metabolites was performed to elucidate the differences in the metabolic pathways between the DIS group and the CON group and between the SI group and the INF group. The 189 differentially abundant metabolites identified between the DIS group and the CON group were subjected to KEGG pathway analysis, which revealed significant differences (*P* < 0.05) in phenylalanine, tyrosine, and tryptophan biosynthesis; phenylalanine metabolism; and porphyrin metabolism between the two groups (Figure 8A). Moreover, KEGG pathway analysis of the 137 different metabolites between the SI group and the INF group revealed significant differences (*P* < 0.05) in phenylalanine, tyrosine, and tryptophan biosynthesis; phenylalanine metabolism; porphyrin metabolism; glycine, serine, and threonine metabolism; and cysteine and methionine metabolism (Figure 8B).

## Discussion

Neonatal sepsis is one of the most common diseases and is reportedly associated with high mortality. Early diagnosis of this condition is crucial for providing timely intervention and improving patient prognosis (Gopal et al., 2023). Moreover, the currently commonly used clinical laboratory tests are of little value in determining the severity of the disease, which is not conducive to the early control of this disease or improvement in terms of the occurrence of complications (Gopal et al., 2023). Therefore, potential biomarkers for the early identification of patients with sepsis and those at high risk for poor prognosis in this disease are urgently needed.

Our study employed untargeted metabolomics technology to analyze peripheral blood metabolites in infants with sepsis and comorbidities, infants with sepsis alone, and infants without any infection. Currently, reports on the metabolomics of infant sepsis are limited, and studies using machine learning to identify the key differentially abundant metabolites for use as biomarkers in the diagnosis of infant sepsis are even rarer. In this research, Hexylamine, psychosine sulfate, LysoPC (18:1 (9Z)/0:0), 2,4,6-tribromophenol, and 25-cinnamoyl-vulgaroside were selected as the diagnostic biomarkers for infant sepsis. Moreover, 12-oxo-20-trihydroxy-leukotriene B4, dihydrovaltrate, PA (8:0/12:0), and 2-heptanethiol were recognized as biomarkers that could identify the presence or absence of complications.

In our study, the levels of hexylamine, psychosine sulfate, LysoPC (18:1 (9Z)/0:0), and 25-cinnamoyl-vulgaroside were significantly lower in the infected children than in the uninfected children, conversely, the level of 2,4,6-tribromophenol was notably higher in infected children. Hexylamine is a monoalkylamine that, as a secondary metabolite of biogenic amines, is involved in the metabolism of amine hormones. When an infection occurs in the body, the inflammatory and immune systems produce a variety of molecules that can bind to receptors on the surface of various types of central nervous system cells, thereby regulating the secretion of norepinephrine by the sympathetic nervous system and of epinephrine by the adrenal system (Thoppil et al., 2023). Previous studies have established that bacterial infections result in increased sympathetic nerve activity and elevated circulating levels of norepinephrine in the body (Kohm and Sanders, 2001). Thus, we hypothesize that hexylamine, as a raw material for the synthesis of amine hormones, is required for the synthesis of norepinephrine and epinephrine, which are elevated after infection, and that this is reflected in a decrease in peripheral serum hexylamine levels in infected children. However, there are few reports on hexylamine as a metabolite, and its role in the regulation of inflammation and the immune system has not been specifically reported. Psychosine sulfate is a derivative of psychosine and belongs to the glycosphingolipids (GLS) class of metabolites. Galactosylceramide (GalCer) and glucosylceramide (GlcCer) are precursors of GLS that are synthesized in the Golgi apparatus under the catalytic action of a series of transferase enzymes, such as galactosyltransferases. In the metabolic pathway of GLS, ceramide, ceramide-1-phosphate, and sphingosine have been shown to be involved in several cellular life activities, including cell proliferation, differentiation, senescence, and apoptosis. Additionally, GLS itself can participate in cellular physiological functions as a binding agent for antigens and microbial toxins (Lahiri and Futerman, 2007). However, there is currently a paucity of research elucidating the specific mechanisms by which glycosphingolipids (GLS) undergo changes in sepsis. In clinical studies, plasma levels of sphingosine-1-phosphate (S1P) were reduced, and ceramide concentrations were increased in adult patients who developed sepsis, indicating abnormalities in sphingolipid and GLS metabolic pathways (Wu et al., 2019). Ceramides activate caspase-dependent apoptotic pathways by forming cell membrane platforms to receive and amplify apoptosis-related signals or by directly interacting with mitochondria (Siskind et al., 2006) and S1P is involved in cell proliferation and antagonizes ceramide-promoted apoptosis (Ogretmen, 2018). Lysophosphatidylcholine (LysoPC) binds primarily to plasma albumin, and peripheral blood albumin levels and LysoPC levels are reduced during severe infections in the body (van de Wouw and Joles, 2022). This finding is consistent with our observation of reduced levels of LysoPC (18:1 (9Z)/0:0) in the peripheral blood serum of infected children. Reduced serum LysoPC has been found in both patients and mouse models of sepsis in previous studies, and even LysoPC has been shown to be strongly associated with patient survival and mortality (Ahn et al., 2017; Lee et al., 2020; Liang et al., 2023). The synthesis and metabolic network of LysoPC are extremely complex, and the exact mechanism for its reduction is not clear at this time, but it is hypothesized to be related to the changes in several enzyme activities found in severe infections, such as secretory phospholipase A2 (SPLA2), cholesterol acyltransferase (LCAT), and lysophosphatidylcholine acyltransferase (LPCAT) (Ahn et al., 2017). The presence of 2,4,6-tribromophenol and 25-cinnamoyl-vulgaroside in sepsis has not been reported; 2,4,6-tribromophenol is mostly considered a toxicant, and the peripheral serum of infected children in this study was significantly upregulated by 2,4,6-tribromophenol.

When comparing children with and without comorbidities, it was hypothesized that the presence of comorbidities indicated a higher disease severity and a further intense inflammatory response. Among the four key differentially abundant metabolites identified in the present study, 12-oxo-20-trihydroxy-leukotriene B4 is a derivative of arachidonic acid and leukotriene metabolism. Leukotrienes are lipid mediators synthesized from arachidonic acid via the 5-lipoxygenase pathway, are produced primarily in leukocytes and are reported to be closely associated with leukocyte activation (Yokomizo, 2015). Leukotriene B4 (LTB4) is an active chemoreceptor, particularly for granulocytes and phagocytes. LTB4 has been implicated in various functions, including neutrophil stimulation and activation, increased interleukin-6 production, and early gene transcription in monocytes (Yokomizo, 2011). In the present study, significantly elevated levels of 12-oxo-20-trihydroxy-leukotriene B4 were observed in children with comorbidities, which was attributed to more severe infections and the heightened metabolism of arachidonic acid and leukotrienes among these children, leading to higher concentrations of these metabolites. Phosphatidic acid (PA) is a glycerophospholipid that forms a pivotal constituent of the lipid bilayer in biofilms and is involved in crucial biological processes such as cellular metabolism and signal transduction (Yang et al., 2018). *In vivo*, PA is involved in an interconversion reaction with lysophosphatidic acid (LPA), which is reported to be closely associated with macrophage migration and infiltration (Jiang et al., 2023). Animal studies have revealed a significant elevation in PA serum levels in mice with sepsis compared to those in control mice, while Wang et al. reported an increased level of peripheral blood PA in infants with sepsis (Ahn et al., 2018; Wang et al., 2023). The present study revealed that children with comorbidities had higher levels of PA than those without comorbidities.

The metabolic pathway analysis conducted in the present study revealed metabolic alterations between infected children and children without any infection, between infected children with complications and those without complications. Interestingly, phenylalanine, tyrosine, and tryptophan biosynthesis, phenylalanine metabolism, and porphyrin metabolism exhibited differences in both analyses, suggesting the potential roles of these pathways in the pathogenesis of infant sepsis. First, it has been reported that phenylalanine, tyrosine and tryptophan biosynthesis and phenylalanine metabolic pathways are altered in sepsis (Li et al., 2023). Phenylalanine, tyrosine and tryptophan are aromatic amino acids, and in the study of Chen et al., major metabolic intermediates of aromatic amino acids, such as phenylpyruvic acid, dopamine and homogentisate, were significantly upregulated in septic patients (Chen et al., 2022). This may be due to inadequate energy supply, insulin resistance, the accumulation of reactive oxygen species (ROS) and muscle tissue breakdown at the onset of sepsis. ROS can reduce the activity of 5,6,7,8-tetrahydrobiopterin (BH4), which serves as a hydrogen donor, thereby affecting the metabolic process of phenylalanine catalyzed by phenylalanine hydroxylase. As a result, this manifests as an accumulation of phenylalanine in the peripheral blood. Additionally, during the occurrence of sepsis, a significant amount of amines such as glucosamine are consumed to synthesize adrenaline, leading to a reduction in small molecule amines. This phenomenon is also discussed in our research (Huang et al., 2019; Feng et al., 2024). Previous studies have shown that aromatic amino acids can compete with branched-chain amino acids to cross the blood‒brain barrier and form pseudo-neurotransmitters that can affect the central nervous system. Due to the downregulation of phenylalanine hydroxylase and tyrosine hydroxylase activities during sepsis, the conversion of phenylalanine and tyrosine to dopamine is blocked, and more phenylalanine and tyrosine may be converted to pseudo-neurotransmitters in the brain, affecting nerve signaling (Gelenberg et al., 1982; Brummelte et al., 2017). The kynurenine pathway, a major pathway of tryptophan catabolism, has been found to be activated during the onset of neuroinflammation. The important role played by the tryptophan kynurenine metabolic pathway in sepsis-associated encephalopathy may be attributed to the fact that increased inflammatory factors activate indoleamine 2,3-dioxygenase (IDO), which metabolizes and produces several neurotoxic factors, such as quinolinic acid (QA) and 3-hydroxykynurenine (3-HAA) (Stone and Darlington, 2013). IDO is thought to be critical for tryptophan metabolism to cause cognitive deficits in septic encephalopathy, and in the study by Gao et al., L-kynurenine, a metabolite of IDO, induced cognitive deficits similar to those in septic mice after injection; these deficits were subsequently relatively ameliorated by administration of the inhibitor of IDO, 1-methyl-D, L-tryptophan (Gao et al., 2016). Attention to the biosynthesis and metabolism of phenylalanine, tyrosine, and tryptophan may help to decipher the mechanism underlying the development of sepsis-associated encephalopathy. In the porphyrin metabolic pathway, porphyrin compounds are primarily synthesized in the liver and can participate in the synthesis and metabolism of heme in the human body (Arora et al., 2016). Current research has shown that during the occurrence and development of sepsis, red blood cells undergo deformation driven and induced by cytokines and pathogens, thereby significantly increasing the level of free hemoglobin in the plasma of septic patients. A higher level of free heme is correlated with the death of patients, and the impaired synthesis and metabolism of heme may affect the porphyrin metabolic pathway (Janz et al., 2013; Jung et al., 2015). Thus, it is hypothesized that alterations in the porphyrin metabolic pathway may be a consequence of red blood cell destruction and heme metabolism during the onset of sepsis. These metabolic changes are, therefore, important indicators of sepsis onset and severity in infants. Accordingly, correcting these metabolic changes could have implications for the treatment of neonatal sepsis.

In summary, by integrating metabolomics with machine learning, we have identified five metabolites that serve as potential biomarkers for the diagnosis of neonatal sepsis, as well as four additional metabolites that may aid in the early differentiation of patients at risk for developing complications. However, our study has certain limitations. Firstly, it is a single-center, small-sample exploratory study with inherent biases. Secondly, the requirements for sample quality in metabolomics analysis are stringent, and although literature suggests that one freeze-thaw cycle may have minimal impact on sample analysis (Goodman et al., 2021), this remains an area of uncertainty for us. Future studies should aim to increase the sample size and expedite metabolomics analysis after sample collection to address these concerns.

## Conclusion

A metabolomics analysis was conducted using infant sepsis serum samples and LC/MS technology, and after univariate data analysis and OPLS-DA, the comparison of infected and noninfected children using LASSO and SVM-RFE revealed five endogenous metabolites that could be used as discriminating biomarkers for neonatal sepsis. The combined AUC of these markers was 1. Four endogenous metabolites were identified by applying the random forest algorithm for discriminating between children with comorbidities and those without comorbidities, and all four markers exhibited excellent discriminatory value. In addition, pathway analysis was conducted, which provided a novel perspective for monitoring infection and determining disease severity in neonatal sepsis patients.

## Data Availability

The raw data supporting the conclusions of this article will be made available by the authors, without undue reservation.
